# TREM2 supports neuronal protection and microglial reactivity without an effect on misfolded protein deposition in chronic neurodegenerative prion disease

**DOI:** 10.3389/fnins.2025.1525017

**Published:** 2025-05-07

**Authors:** Sarah M. Carpanini, Barry M. Bradford, Alessio Alfieri, Pedro Piccardo, Jimena Monzón-Sandoval, Deborah Brown, Aileen Boyle, Aleksandra Pokrovskaya, Neil A. Mabbott, Jean Manson, Barry W. McColl

**Affiliations:** ^1^UK Dementia Research Institute, Cardiff University, Cardiff, United Kingdom; ^2^The Roslin Institute & Royal (Dick) School of Veterinary Studies, University of Edinburgh, Midlothian, United Kingdom; ^3^Centre for Discovery Brain Sciences, University of Edinburgh, Edinburgh, United Kingdom; ^4^UK Dementia Research Institute, Edinburgh Medical School, Centre for Discovery Brain Sciences, University of Edinburgh, Edinburgh, United Kingdom

**Keywords:** TREM2, neurodegenerative disease, microglial reactivity, pathology, prion disease, neuroinflammation

## Abstract

**Introduction:**

Triggering receptor expressed on myeloid cells-2 (*TREM2*) variants have been identified as risk factors for neurodegenerative disease, including Alzheimer’s disease. TREM2 is a cell surface receptor on microglia that regulates homeostatic and immunomodulatory functions, including phagocytosis of apoptotic debris and the resolution of damage-associated inflammation. It remains unclear how TREM2 may mediate an influence on neurodegenerative disease, particularly in relation to key neuropathological hallmarks such as neuronal loss and proteinopathy.

**Methods:**

We used the ME7 prion disease model to assess the role of TREM2 in the progression and pathology of neurodegenerative disease. Prion diseases are characterised by the accumulation of misfolded prion proteins and provide a highly tractable platform to determine if TREM2 has disease-modifying effects.

**Results:**

*Trem2*^−/−^ and wild-type (WT) mice were inoculated intracerebrally with mouse-passaged ME7 scrapie prions, and their effects on CNS disease pathogenesis were determined. Although the accumulation of prion disease-specific PrP was similar in the brains of mice from each group, the severity of neuropathology was increased in *Trem2*^−/−^ mice. Morphometric analysis of the microglia also indicated blunted disease-induced reactivity in the brains of infected *Trem2^−/−^* mice compared to wild-type (WT) controls. Expression of genes involved in myelination was reduced in prion-infected *Trem2^−/−^* mice compared to infected WT mice.

**Conclusion:**

We conclude that during brain infection with prions, TREM2 supports microglial reactive changes associated with resilience to neuronal loss independently of affecting misfolded PrP deposition. These data imply that TREM2 status may be an important influence on the downstream response to CNS proteinopathy, which alters the susceptibility of neurons and brain tissue to proteinopathy-induced degenerative changes.

## 1 Introduction

Neurodegenerative diseases are one of the largest public health risks in the developed world, bringing both social and economic costs to the individual and society as a whole (Feigin and Vos, 2019). Despite widespread research, our understanding of the causative effects leading to neurodegeneration is limited. Several studies have implicated the role of inflammation in the pathogenesis of neurodegenerative disease, including the altered function of microglia (Aguzzi and Zhu, 2017; Yeh et al., 2017). Microglia function as the primary innate immune cells of the central nervous system (CNS) parenchyma, displaying a wide array of roles during development, homeostasis, and disease, including clearance of cellular debris and apoptotic cells. Microglia are highly dynamic; under healthy conditions, microglia demonstrate a ramified morphological phenotype with long processes extending out and surveying the local environment. Microglia commonly become reactive upon injury, infection, inflammation, and degeneration, which involves alterations to their morphology, transcriptional activity, and proteo-metabolic profiles (Paolicelli et al., 2022). How microglia influence the progression of neurodegenerative disease is currently an area of intense focus (Bradford et al., 2022; Pons et al., 2021).

Triggering receptor expressed on myeloid cells-2 (TREM2) is a cell surface transmembrane glycoprotein and is expressed on a subset of myeloid cells, including dendritic cells and tissue-specific macrophages. In the CNS parenchyma, TREM2 is exclusively expressed in microglia (Takahashi et al., 2005; Sessa et al., 2004; Kiialainen et al., 2005). Within microglia, TREM2 plays a critical role in immune surveillance and regulation of homeostatic and immunomodulatory functions, such as phagocytosis of apoptotic debris and the resolution of damage-associated inflammation (Takahashi et al., 2005). An important role of TREM2 in regulating microglia responses in humans is implicated by its association with certain genetic diseases. Homozygous mutations in *TYROBP* (encoding the TREM2 ligand DAP12) or *TREM2* cause Nasu–Hakola disease associated with progressive, early-onset dementia, bone cysts, and demyelinating lesions in the CNS (Paloneva et al., 2002). Individuals heterozygous for rare inactivating variants of *TREM2*, notably the *R47H* mutation, are at increased risk of developing Alzheimer’s disease (AD) (Pottier et al., 2013). Additionally, studies have shown that TREM2 expression is upregulated upon inflammatory insult and/or neurodegenerative disease, including Parkinson’s disease (PD) (Liu et al., 2016), amyotrophic lateral sclerosis (ALS) (Cady et al., 2014), stroke (Kawabori et al., 2015), traumatic brain injury (Saber et al., 2017), and AD (Lue et al., 2015).

Loss of TREM2 has been shown to have detrimental effects on the pathology and outcome of acute injuries, such as ischemic stroke (Kawabori et al., 2015), and in cuprizone-induced demyelinating models (Cantoni et al., 2015; Nugent et al., 2020; Poliani et al., 2015). The crossbreeding of *Trem2^−/−^* mice to models of Alzheimer’s disease-related proteinopathy has resulted in inconsistent findings that may reflect disease stage-dependent effects. Beneficial and detrimental disease phase-associated effects have been observed in both tauopathy (Leyns et al., 2017; Vautheny et al., 2021; Bemiller et al., 2017) and amyloidopathy (Jay et al., 2015; Meilandt et al., 2020; Jay et al., 2017) models.

A limitation of the majority of chronic proteinopathy models is a lack of overt neuronal pathology or neuronal loss, so it is challenging to assess the links between proteinopathy and neurodegeneration that are characteristic of human disease. In prion-induced neurodegeneration, the precise time course of disease pathology and clinical endpoints is well-characterised. These include deposition of the misfolded prion protein (PrP), marked neuronal and synapse loss, vacuolation, and gliosis (Bradford et al., 2022), thus providing a robust platform to determine if TREM2 has a disease-modifying effect on key elements of a neurodegenerative process.

A previous study reported a lower density of microglia in mice infected with the Rocky Mountain Laboratories (RML) prion isolate and no difference in prion accumulation in the brains (Zhu et al., 2015). Our study is consistent with and expands on this study (Zhu et al., 2015) by using an alternative prion agent and including assessment of glial and neuropathological changes alongside transcriptome-wide analysis of brain tissue at an early stage of the disease. We utilised the mouse-passaged ME7 scrapie strain to provide further insight into the role of TREM2 in disease pathogenesis. Importantly, microglial reactivity is well-established to occur early in the pathogenesis of disease (Betmouni et al., 1996). We set out to establish whether loss of TREM2 had any disease-modifying effects on the progression of prion disease. We show that the loss of TREM2 aggravated neuronal loss and vacuolation, which was associated with blunted microglial reactivity occurring without alterations to PrP deposition, astrogliosis, or clinical endpoints. Transcriptomic profiling identified TREM2-dependent regulation of pathways associated with microglial reactivity and myelination at an early phase of the disease. TREM2 may promote microglial reactive responses and resilience to degeneration during prion infection.

## 2 Materials and methods

### 2.1 Animal husbandry and genotyping

All procedures involving live animals were carried out under the authority of a UK Home Office Project Licence in accordance with the ‘Animals (Scientific Procedures) Act 1986’ and Directive 2010/63/EU and were approved by The University of Edinburgh Bioresearch & Veterinary Services Animal Welfare and Ethics Review Body (AWERB). *Trem2*^tm1(KOMP)Vlcg^ mice (*Trem2^−/−^*) that do not express TREM2 protein on a C57BL/6NTac background were produced at the University of California-Davis and re-derived at MRC Harwell (Oxford, UK). C57BL/6NTac mice were used as wild-type controls. Animals were maintained in a standard 12-h light-12-h dark cycles and given access to food and water *ad libitum*.

For genotyping, DNA was extracted from ear notch tissue (collected at weaning) using Qiagen DNeasy blood and tissue kit following the manufacturer’s protocol (Qiagen, Crawley, United Kingdom). All animals were genotyped for the presence or absence of the gene trap cassette using Jax MegaMix Blue Taq Polymerase.

Primer sequences for genotyping were as follows:

wild-type forward- GACTCGTTAAGACACTGCCAAGAGC,

wild-type reverse- CCTCTTCTCCTACCTGGGTTTGTCC,

*Trem2* forward- GCAGCCTCTGTTCCACATACACTTCA

*Trem2* reverse- ATCTCAGACTGCATTCTCCCACTCC.

PCR cycles were as follows: 95°C for 3 min, followed by 30 cycles of 95°C for 30 s, 60°C for 30 s, and 72°C for 45 s. A final extension phase of 67°C for 10 min was performed, and samples were subsequently stored at 4°C. PCR products were run on QIAxcel (Qiagen, Crawley, United Kingdom) and imaged.

### 2.2 Prion inoculum and challenge

Animals aged 7–13 weeks (mixed sex) were inoculated intracerebrally with 20 μL of a 1% (w/v) brain homogenate obtained from mice terminally infected with the mouse-passaged ME7 scrapie prion strain. Control mice were instead injected with normal brain homogenate (NBH). Both sexes were used for clinical end-stage experiments, whereas only females were studied 90-days post-inoculation.

Mice were monitored daily and phenotypically scored weekly from 50-days post-inoculation for clinical signs of disease as previously published (Brown and Mabbott, 2014). The scorer was blind to the genotype and treatment groups. Mice were terminated 90-days post-inoculation (*N* ≥ 5 prion-infected, *N* ≥ 3 NBH) or at the clinical end-stage of the disease (*N* = 7 prion-infected, *N* = 3 NBH) as described previously (Brown and Mabbott, 2014). Survival times were defined as the time (in days) between inoculation and termination.

### 2.3 Tissue preparation

Animals were culled humanely by transcardiac perfusion with physiological saline. Brains were removed and halved sagittally. One hemisphere was fixed for 48 h in 10% formal saline and paraffin-embedded for histopathology. The other hemisphere was snap-frozen in liquid nitrogen and stored at −80°C for microarray analysis. Paraffin-embedded brain tissue was dehydrated in ethanol and embedded in paraffin. Coronal sections were cut at 6 μm using a microtome and mounted on superfrost slides.

### 2.4 Vacuolation scoring

Paraffin-embedded brain tissue was dewaxed by immersing it in xylene and re-hydrated through a series of decreasing alcohol concentrations. Tissue sections were stained with haematoxylin and eosin for neuropathologic evaluation (Fraser and Dickinson, 1967). Nine regions of the grey matter, 1, dorsal medulla; 2, cerebellar cortex; 3, superior colliculus; 4, hypothalamus; 5, thalamus; 6, hippocampus; 7, septum; 8, retrosplenial and adjacent motor cortex; 9, cingulate and adjacent motor cortex, were scored on a scale of 0 (no vacuolation) to 5 (severe vacuolation), and three regions of white matter; 10, cerebellar white matter; 11, midbrain white matter; and 12, pyramidal tract at the level of the thalamus were scored on a scale of 0 (no vacuolation) to 3 (severe vacuolation). The neuropathology analysis was performed by an observer blinded to the experimental design. The mean score for each experimental group was calculated and plotted to produce the lesion profile.

### 2.5 Immunohistochemistry

Brain sections were deparaffinised and re-hydrated through a series of alcohol concentrations. For PrP, immunostaining slides were immersed in a citric acid buffer (0.1 M citric acid, 0.1 M sodium citrate, pH 6.4) and autoclaved at 121°C for 15 min. Slides were cooled in running water, followed by immersion in 98% formic acid for 10 min. Endogenous peroxidase was blocked by the immersion of tissue sections in 1% H_2_O_2_/methanol and normal goat serum. Tissue sections were incubated overnight with anti-PrP antibody 6H4 (1:500; Life Technologies (cat no. 7500996)). Antibody binding was detected using biotinylated species-specific secondary antibodies (Jackson Laboratory) and Vectastain Elite ABC Kit (Vector Laboratories); peroxidase activity was visualised using 3,3′-diaminobenzidine (DAB). All slides were counterstained with haematoxylin.

For immunohistochemical detection of microglia and astrocytes, deparaffinised and re-hydrated brain sections were either given no antigen retrieval step [glial fibrillary acidic protein (GFAP)] or were immersed for 10 min in 10 mM citrate buffer [anti-ionised calcium binding adaptor molecule 1 (IBA1), also known as allograph inhibitory factor-1 (AIF-1)], and microwaved for 10 min. Tissue sections were incubated for 1 h at room temperature with anti-GFAP (1:400, DAKO Z0334) to detect astrocytes. Anti-IBA1 antibody (1:1000, WAKO 019–9,741) was used to identify microglia. Antibody binding was detected using biotinylated species-specific secondary antibodies (Jackson Laboratory) and Vectastain Elite ABC Kit (Vector Laboratories). Peroxidase activity was visualised using DAB. Sections were counterstained with haematoxylin.

Brain sections from prion-inoculated animals probed with secondary antibodies (in the absence of primary antibodies) served as negative controls in all experiments.

For assessment of myelin, deparaffinised brain sections were stained overnight in 0.1% Luxol Fast Blue at 55°C and differentiated in 0.05% Lithium Carbonate.

### 2.6 Image analysis and quantification of immunostaining

Tissue sections were assessed by an observer blinded to genotype and treatment groups. The following brain regions were scored from samples taken 90-days post-inoculation (dpi): hippocampus, thalamus, brainstem, and cortex. For clinical end-stage experiments, two layers of the cortex, caudate, septum, hippocampus, thalamus, hypothalamus, brainstem-pons, brainstem-medulla, cerebellum-nuclei, and cerebellum-cortex were scored for all experiments.

For PrP-stained slides, individual brain regions were scored as follows: 0 (no PrP accumulation), 1 (mild), 2 (moderate), and 3 (severe) for the entire brain region, and the deposition of fine-punctate, coarse, and or plaque-like deposits was noted. TBH immunised animals should always score 0. For IBA1 semi-quantitative analysis, IBA1 immunoreactive cells were evaluated according to the following morphological characteristics: 0 (ramified), 1 (mild amount of ameboid positive cells), 2 (moderate amount of ameboid positive cells), and 3 (large amount of ameboid cells). For IBA1 and GFAP quantitative analysis, image analysis was performed using ImageJ software.^1^ In brief, the optical density (OD) values for immunostaining were calculated using ImageJ software following H-DAB deconvolution. Mean grey OD values were measured from DAB greyscale images (scaled 0–255) and expressed as a % relative intensity by dividing by the maximum value (255). Deconvoluted images were subjected to thresholding, and the % area coverage or distribution of positive DAB immunostaining was measured using ImageJ. Finally, deconvoluted and threshold images were subjected to particle analysis with a minimum size exclusion of <50 μm2 to determine the GFAP+ object (astrocyte) or IBA1+ (microglia) average size. Hippocampal CA1 pyramidal neuron cell density was assessed on haematoxylin and eosin-stained sections imaged using an x10 objective lens; in brief, the total number of neuronal somas was counted in Fiji (ImageJ) using Cell Counter, and density was calculated by dividing the total number of neuronal soma by the length of the CA1 pyramidal layer.

### 2.7 RNA extraction

RNA was extracted using the miRNeasy mini kit (Qiagen). The kit was carried out according to the manufacturer’s instructions, with the final RNA elution repeated twice in 50 μL RNase-free water. RNA concentration was quantified using the Thermo Scientific ND 1000 Nanodrop. Female animals only at 90-days post-inoculation were used for microarray analysis.

### 2.8 Microarray processing

A total of 14 samples were used for microarray analysis (Affymetrix platform HT MG-430 PM). Gene expression microarrays were performed by Edinburgh Genomics, University of Edinburgh. Scanned images (DAT files) were transformed into intensities (CEL files) using AGCC software (Affymetrix). Initial quality control was performed using the *arrayQualityMetrics* package from Bioconductor (Kauffmann et al., 2009). Intensities for 45,141 probes were normalised using the Robust Multiarray Average algorithm implemented in the *affy* package from Bioconductor (Gautier et al., 2004). We filtered out probes without RMA-normalised intensities greater than 4 in at least two samples. Probe to gene annotations were retrieved from the Affymetrix website.^2^ RMA normalised intensities were aggregated to gene level using the mean, obtaining relative gene expression values for 12,758 genes across 14 samples. Principal component analysis was performed using the *prcomp* function in R.

### 2.9 Differential expression and gene ontology analysis

Differential expression analysis was performed using the *limma* package from Bioconductor (Ritchie et al., 2015). We adjusted for multiple tests using the Benjamini and Hochberg method. We performed gene ontology enrichment analysis using the *clusterProfiler* package from Bioconductor (Yu et al., 2012). We used the genome-wide annotations from mice “org.Mm.eg.db,” and we used the genes detected in the array as background population. We performed GO enrichment analysis both on the population of DEGs with an adjusted *p*-value <0.05 and then on the top DEGs with a nominal p-value of <0.005. Gene network visualisation was performed using Cytoscape (release 3.8.1).

### 2.10 Protein–protein interaction network

Combined protein scores for protein–protein links were downloaded from the STRING website for *Mus musculus* (10,090.protein.links.v11.0) (Von Mering et al., 2005). Protein Ensembl IDs were mapped first to Gene Ensembl IDs and then to Mouse Genome Informatics symbols using annotations downloaded from Ensembl biomart (release 104). Protein–protein interaction (PPI) network was reduced to the gene level; if more than one protein–protein interaction exists for the corresponding gene pairs, we have calculated the average combined score. We tested if there were more PPIs than expected by chance amongst sets of DEGs (nominal *p*-value <0.005), based on 10,000 randomisations, whilst controlling for the tissue-specific gene background population, Coding DNA sequences length, and a degree in the network. CDS length was also obtained from Ensembl BioMart, annotated to MGI symbols, where we have taken the maximum known CDS per gene. Similarly, we also tested if the sum of the combined scores was higher than expected by chance.

### 2.11 Statistical analysis

Statistical analysis was performed using GraphPad Prism version 10. Sample sizes were not determined formally. The Shapiro–Wilk test was used to check for normal distribution in all datasets. The log-rank (Mantel–Cox) test was used to compare survival to clinical end-stage. Two-way ANOVA with Sidak’s multiple comparison test was used to compare genotypes in lesion profile analysis. Two-way ANOVA with Bonferroni multiple correction was used to compare vacuolation profiles. Two-way ANOVA with Sidak’s multiple comparisons was used in GFAP, IBA1, and neuronal density analysis. Results are presented as the mean ± standard error of the mean (SEM).

## 3 Results

### 3.1 *Trem2* deletion aggravates prion disease neuronal pathology

**Figure 1:**
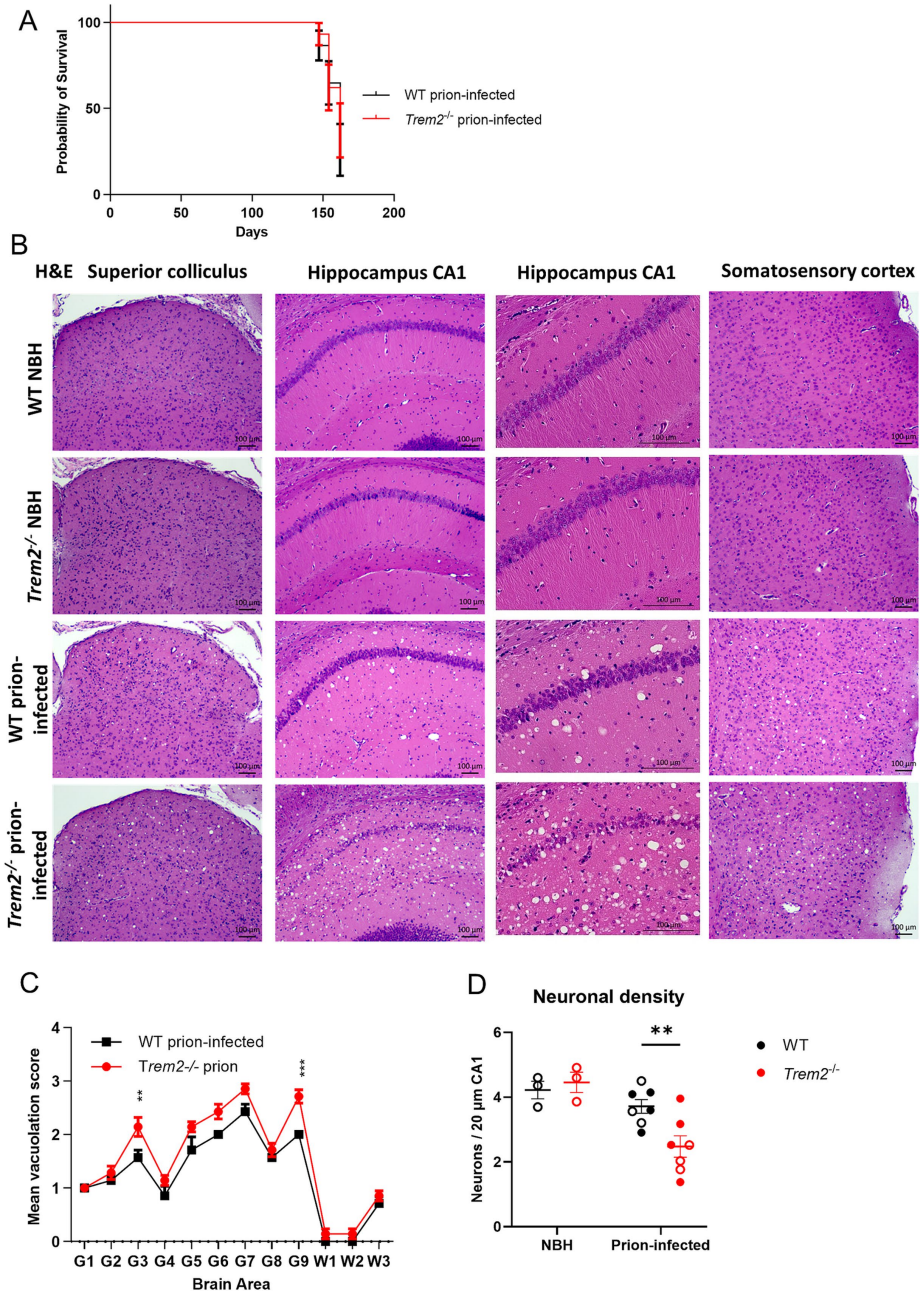
*Trem2^−/−^* mice infected with prion disease show increased vacuolation and neuronal loss but no overall impact on survival. **(A)** Kaplan–Meier survival curves showing no difference in onset of symptoms or survival between wild-type (WT) and *Trem2* knockout (*Trem2^−/−^*) mice following intracerebral inoculation of prions. Log-rank (Mantel–Cox) test. **(B)** Haematoxylin and eosin-stained brain sections from the superior colliculus, hippocampus CA1, and somatosensory cortex from WT normal brain homogenate (WT NBH), *Trem2^−/−^* NBH, WT prion-infected and *Trem2^−/−^* prion-infected mice; WT NBH N = 3, *Trem2^−/−^* NBH N = 3, WT prion-infected N = 7, and *Trem2^−/−^* prion-infected *N* = 7 mice. **(C)** Lesion profiling in prion-infected WT and *Trem2^−/−^* brains. Vacuolation scoring in grey matter areas (G) of the brain analysed: 1, dorsal medulla; 2, cerebellar cortex; 3, superior colliculus; 4, hypothalamus; 5, thalamus; 6, hippocampus; 7, septum; 8, retrosplenial and adjacent motor cortex; 9, cingulate and adjacent motor cortex. White matter areas (W) analysed: 10, cerebellar white matter; 11, midbrain white matter; 12, pyramidal tract at the level of the thalamus. Two-way ANOVA with Sidak’s multiple comparison test. Scale bars represent 100 μm. ^*^p < 0.05; ^*^p < 0.005, ^**^p < 0.001; ^****^p < 0.0001. **(D)** Measurement of neuronal density in CA1 hippocampus per 20 μm stained with haematoxylin and eosin. Two-way ANOVA with Sidak’s post-hoc test was used to compare neuronal densities; genotype main effect (*p* = 0.154), disease main effect (*p* = 0.002), genotype x disease interaction (*p* = 0.042); **Padj <0.01. Open circles represent females; closed circles represent males. Data show mean ± SEM.

Wild-type (WT) mice and *Trem2^−/−^* mice infected with ME7 scrapie prions showed no significant difference in the onset (142 ± 2.57 dpi WT and 142 ± 2.57 dpi *Trem2^−/−^*) of the clinical signs of prion disease (including kyphosis, lethargy, unkempt hair, and ataxia) or duration to clinical end-stage (154 ± 2.56 dpi WT and 154 ± 1.98 dpi *Trem2^−/−^*) between genotypes (Figure 1A). Prion infection in the brain is characterised by the deposition of abnormal prion protein PrP^Sc^, microgliosis, astrogliosis, neuronal loss, and spongiform abnormalities. Histopathological analysis of brain sections from prion-infected WT and *Trem2^−/−^* mice showed widespread vacuolation in the grey matter at the clinical end-stage of the disease (Figure 1B). A significant increase in the magnitude of the vacuolation was observed in the superior colliculus and forebrain cortex of prion-infected *Trem2^−/−^* mice when compared to prion-infected WT mice (Figure 1C). In contrast, no evidence of vacuolation was observed in age-matched brains from normal brain homogenate (NBH)-injected control mice from each genotype. The vacuolation observed in the brains of prion-infected mice often co-occurs with neuronal perikaryal pathology (Williams et al., 1997). Marked neuronal loss and loss of presynaptic terminals in the CA1 area of the hippocampus of ME7-injected mice have previously been reported (Hilton et al., 2013). However, the cellular integrity of the dentate gyrus is preserved (Gomez-Nicola et al., 2014). Therefore, we focused on an area of established vulnerability in this model. Measurement of neuronal density in the hippocampal CA1 region was assessed on H&E-stained sections and calculated as the number of neurons per 20 μm CA1 and showed significantly lower density in *Trem2^−/−^* prion-infected mice in the absence of any genotype effect at baseline in NBH groups (Figure 1D). In summary, *Trem2* deficiency worsened regional vacuolar pathology and neuronal loss in the prion-infected brain, although it did not affect clinical disease onset or duration.

### 3.2 *Trem2* deletion has no impact on the deposition of prion protein

**Figure 2:**
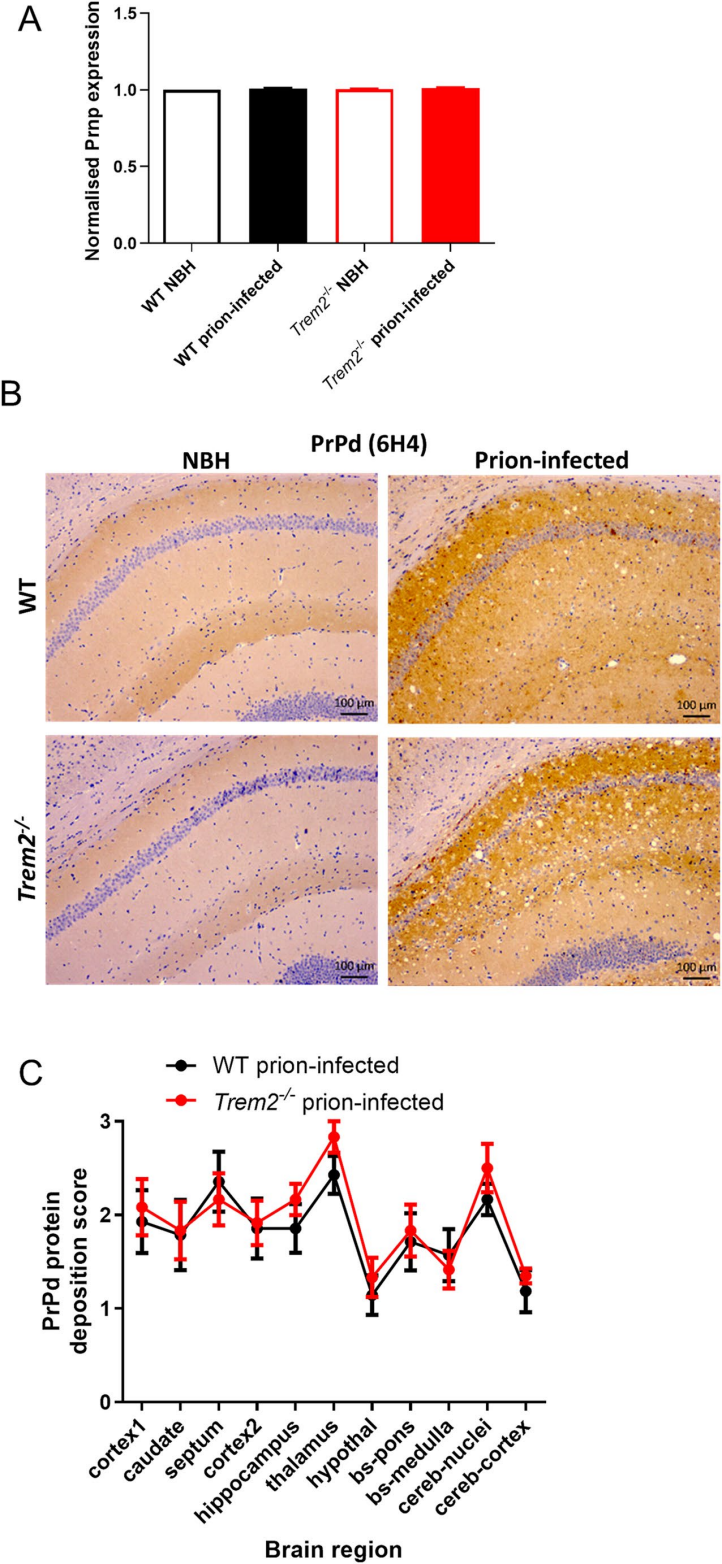
*Trem2^−/−^* mice infected with prion disease show no differences in the amount of prion protein deposited. **(A)** Measurement of *Prnp* gene expression at 90 days post-infection with prion disease or normal brain homogenate (NBH) normalised to WT NBH. **(B)** Prion protein deposition (PrPd) at the clinical endpoint of disease quantified by immunostaining. Images show the hippocampal CA1 region. **(C)** Semi-quantification of PrPd deposition in different brain regions. Results are presented as mean ± standard error of the mean (SEM). Scale bars represent 100 μm.

We then assessed whether loss of *Trem2* has any impact on the accumulation or distribution of disease-specific prion protein (PrP^d^) within the brain. No differences in the expression of *Prnp*, the gene encoding the cellular prion protein (PrP^C^), measured by microarray, were observed in the brains of NBH-injected control mice or prion-infected mice at 90 days post-injection irrespective of genotype (Figure 2A). At the clinical end-stage, all prion-infected mice showed widespread accumulation of PrP throughout the brain irrespective of genotype (Figure 2B). Fine punctate, coarse, and/or plaque-like and pericellular deposits were observed in both WT and *Trem2^−/−^* prion-infected mice at the clinical endpoint. Semi-quantitative analysis of the abundance of the PrP^d^ immunostaining showed that the magnitude of the PrP^d^ accumulation in the brains of prion-infected WT and *Trem2^−/−^* mice was similar (Figure 2C). Together, these data show that *Trem2* deficiency did not influence the accumulation of PrP^d^ in the brains of infected mice.

### 3.3 Microglial reactivity is altered in *Trem2^−/−^* prion-infected mice

**Figure 3:**
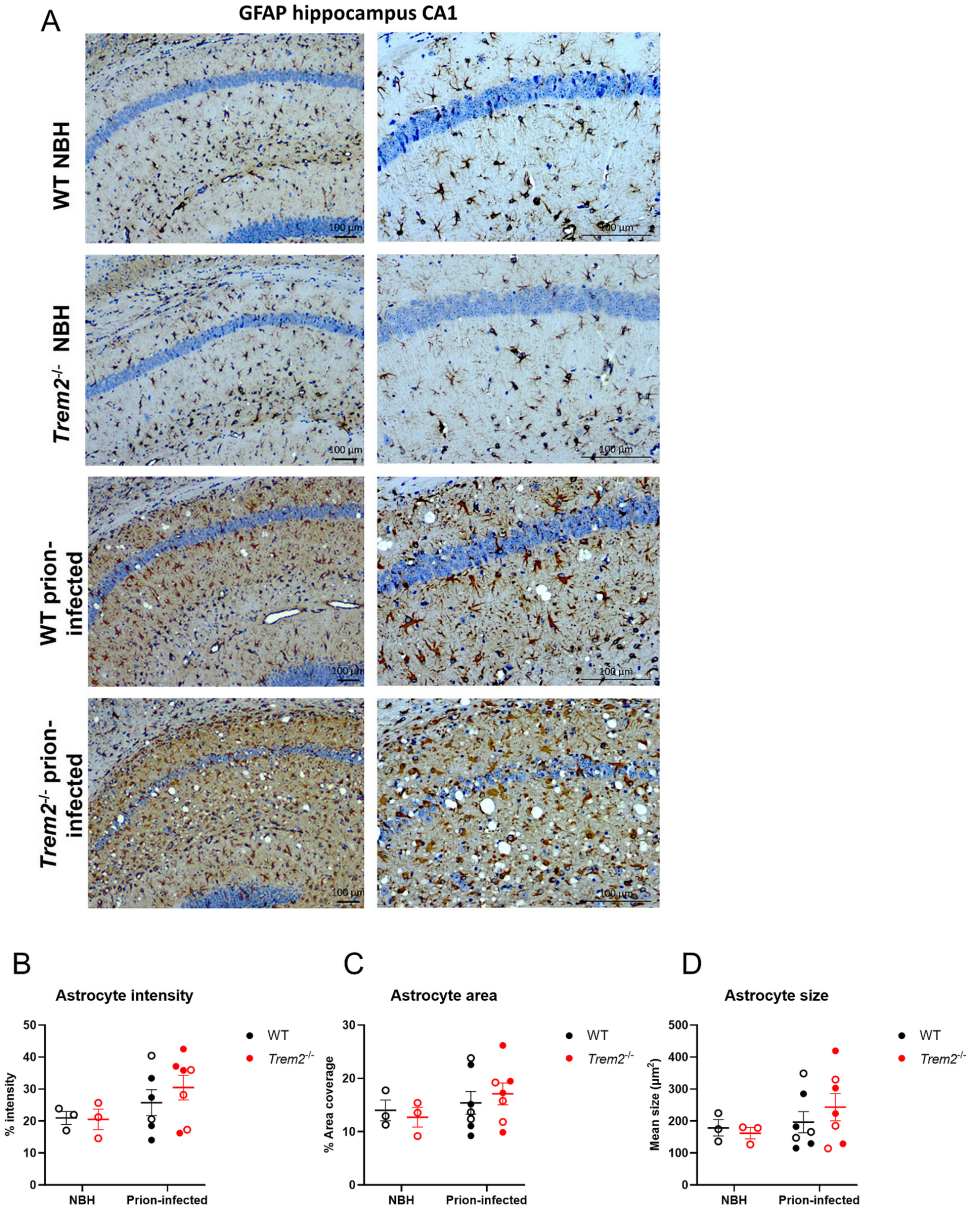
*Trem2^−/−^* mice infected with prion disease show no differences in astrogliosis. **(A)** Astrocyte activation quantified by immunostaining for GFAP in WT normal brain homogenate (WT NBH), *Trem2^−/−^* NBH, WT prion-infected, and *Trem2^−/−^* prion-infected mice. Images show the hippocampus CA1 region. **(B)** Astrocyte percentage intensity; two-way ANOVA with Sidak’s post-hoc test; genotype main effect (*p* = 0.639), disease main effect (*p* = 0.117), genotype x disease interaction (*p* = 0.567). **(C)** Astrocyte percentage area coverage; two-way ANOVA with Sidak’s post-hoc test; genotype main effect (*p* = 0.936), disease main effect (*p* = 0.256), genotype x disease interaction (*p* = 0.554). **(D)** Average astrocyte size; two-way ANOVA with Sidak’s post-hoc test; genotype main effect (*p* = 0.733), disease main effect (*p* = 0.27), genotype x disease interaction (*p* = 0.474). Open circles represent females; closed circles represent males. Results are presented as mean ± SEM. Scale bars represent 100 μm.

Glial reactivity is a common pathological feature during the progression of prion disease (Hilton et al., 2013; Borner et al., 2011; Bradford et al., 2022). We, therefore, examined the brains of WT and *Trem2^−/−^* mice either prion-infected or inoculated with NBH for any alterations in the glial response. Brain sections were immunostained for the reactive astrocyte marker GFAP. GFAP immunostaining was observed in white matter tracts in all animals irrespective of genotype and treatment groups (Figure 3A). No significant main effect of genotype or interaction effect (genotype x disease) was observed in staining intensity, area, or number of astrocytes at the clinical end-stage of the disease (Figures 3B–D), indicating genotype is not modulating these astrocyte features. We do, however, conservatively draw attention to trends for a main effect of disease on elevated astrocyte metrics that may warrant further investigation in larger studies.

**Figure 4:**
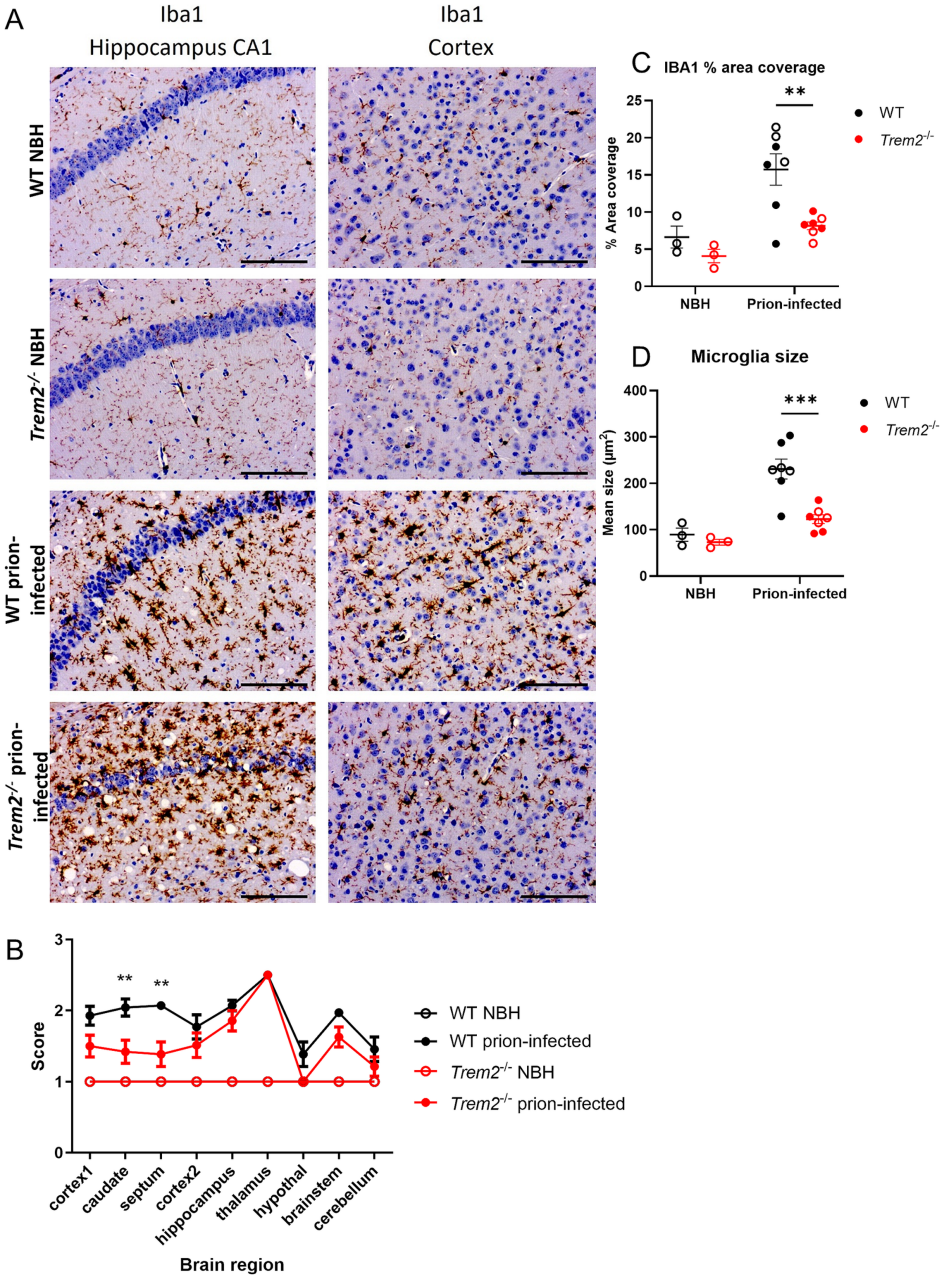
Microglia from *Trem2^−/−^* mice show a less “active” morphology at the endpoint of prion disease. **(A)** IBA1 stained microglia in the hippocampus CA1 region and cortex of WT and *Trem2^−/−^* mice either NBH or prion-infected at the clinical endpoint of disease. **(B)** IBA1 immunoreactive cell morphology in cortex 1, caudate (caudate nucleus), septum, cortex 2, hippocampus, thalamus, hypothalamus (hypothal), brainstem, and cerebellum; two-way ANOVA with Sidak’s multiple comparison test was used to compare between prion-infected genotypes in IBA1 analysis. Scale bars indicate 100 μm. ^*^p < 0.05; ^*^p < 0.005. **(C)** Microglia percentage area coverage; two-way ANOVA with Sidak’s post-hoc test; genotype main effect (*p* = 0.012), disease main effect (*p* = 0.002), genotype x disease interaction (*p* = 0.1775); **Padj <0.01. **(D)** Average microglia size; two-way ANOVA with Sidak’s post-hoc test; genotype main effect (*p* = 0.0051), disease main effect (*p* = 0.0001), genotype x disease interaction (*p* = 0.0292); **Padj <0.001. Open circles represent females; closed circles represent males. Results are presented as mean ± SEM. Scale bars indicate 100 μm.

In the healthy brain, microglia exhibit a ramified morphology. During disease and/or inflammation, including in prion disease (Betmouni et al., 1996), microglia undergo morphological transformation from a ramified to a more amoeboid morphology with an enlarged cell body and shorter, thickened processes. We examined the impact of *Trem2* deletion on microglial morphology by immunostaining for the microglia/macrophage marker IBA1 at the clinical endpoint of the disease. Microglial cells were scored based on their morphology. We observed no differences in microglial morphology between genotypes in the absence of disease, with the majority of microglia examined showing a ramified morphology (Figure 4A) (WT NBH and *Trem2^−/−^* NBH). In the brains of WT prion-infected mice, a more amoeboid microglial morphology was observed, suggesting the microglia were more reactive. However, in the brains of *Trem2^−/−^*prion-infected mice, the microglia typically presented with a ramified (less amoeboid) morphology, specifically in the caudate and septum, and reflected by significant differences in the semi-quantitative microglial scores (Figure 4B). Deeper quantification of microglial features (% area coverage and size) in the vulnerable hippocampal CA1 region substantiated an interaction between prion disease and genotype with significantly less coverage and smaller cell size in prion-infected *Trem2*^−/−^ compared to WT mice (Figures 4C,D). These data suggest altered (blunted) reactivity of microglia in the prion disease state when *Trem2* is deleted.

### 3.4 Transcriptome analysis reveals alterations in *Trem2^−/−^* prion-infected brain at early disease phase

**Figure 5:**
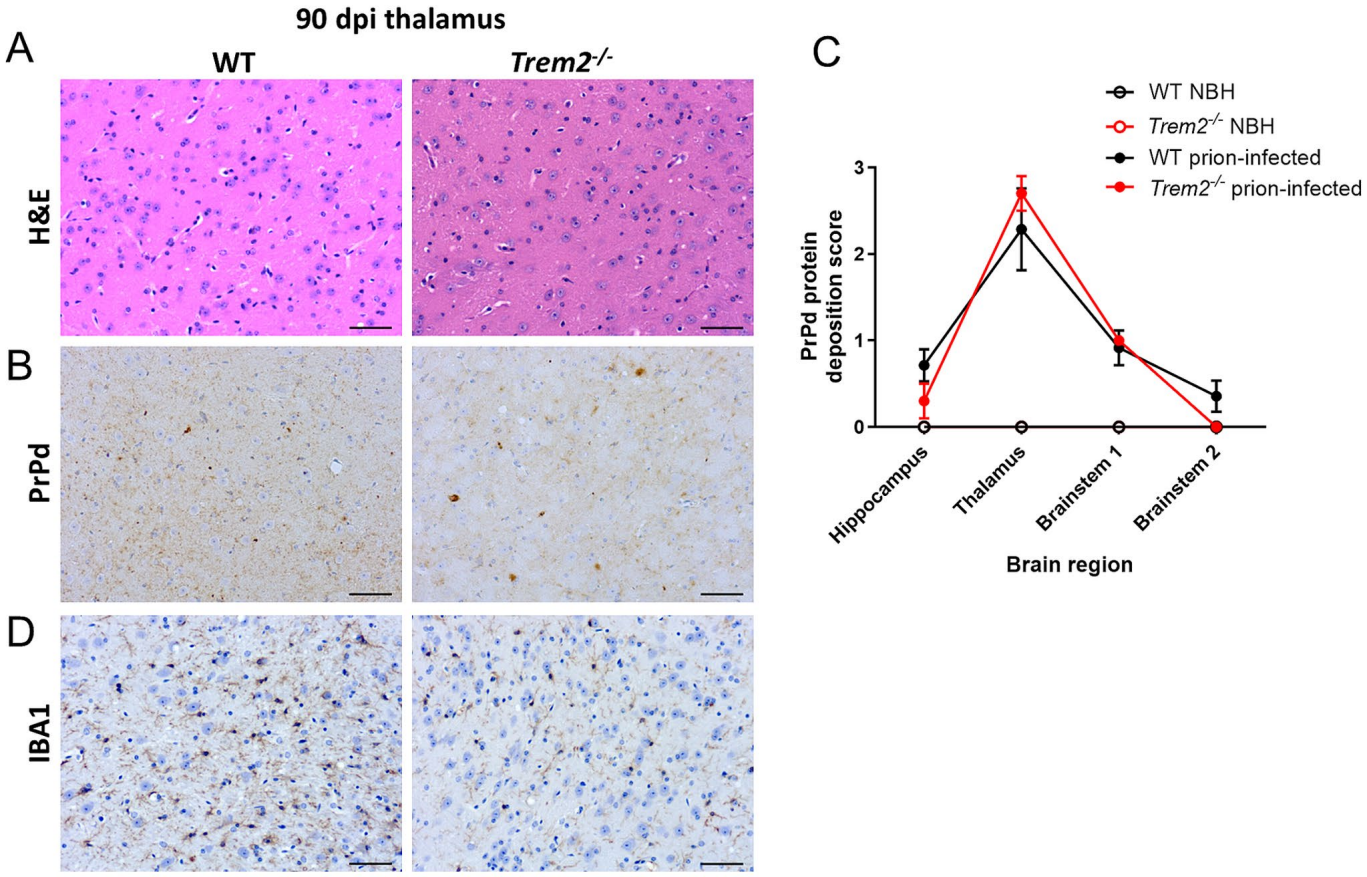
Histopathological analysis showing no differences in pathogenesis or microgliosis observed in *Trem2^−/−^* mice at 90 days post prion infection. Histopathological analysis in wild-type (WT) and *Trem2^−/−^* mice 90 days post prion-infection. **(A)** Haematoylin and eosin (H&E) staining in the thalamus of prion-infected mice. **(B)** Prion protein deposition (PrPd) at 90 dpi quantified by immunostaining in the thalamus. **(C)** Semi-quantification of PrPd deposition in different brain regions; two-way ANOVA with Sidak’s multiple comparison test. **(D)** Analysis of IBA1 immunostaining in the thalamus. Scale bars represent 50 μm. Error bars represent the standard error of the mean (±SEM).

Next, transcriptome analysis by microarray was used to explore candidate mechanisms and identify potential molecular pathways that may contribute to the effects of *Trem2* deficiency on the aggravated neuronal pathology and reduced microglial reactivity observed during CNS prion disease. WT mice and *Trem2^−/−^* mice were injected with prions or NBH as a control, and brains were collected during the pre-clinical phase at 90 days after infection when only limited histopathological signs of prion disease were evident in the brain. At this time point, no significant differences in the magnitude and distribution of the vacuolation (Figure 5A) or PrP^d^ deposition (Figures 5B,C) or microglial morphological state (Figure 5D) were observed between brains from WT mice and *Trem2^−/−^* prion-infected mice.

**Figure 6:**
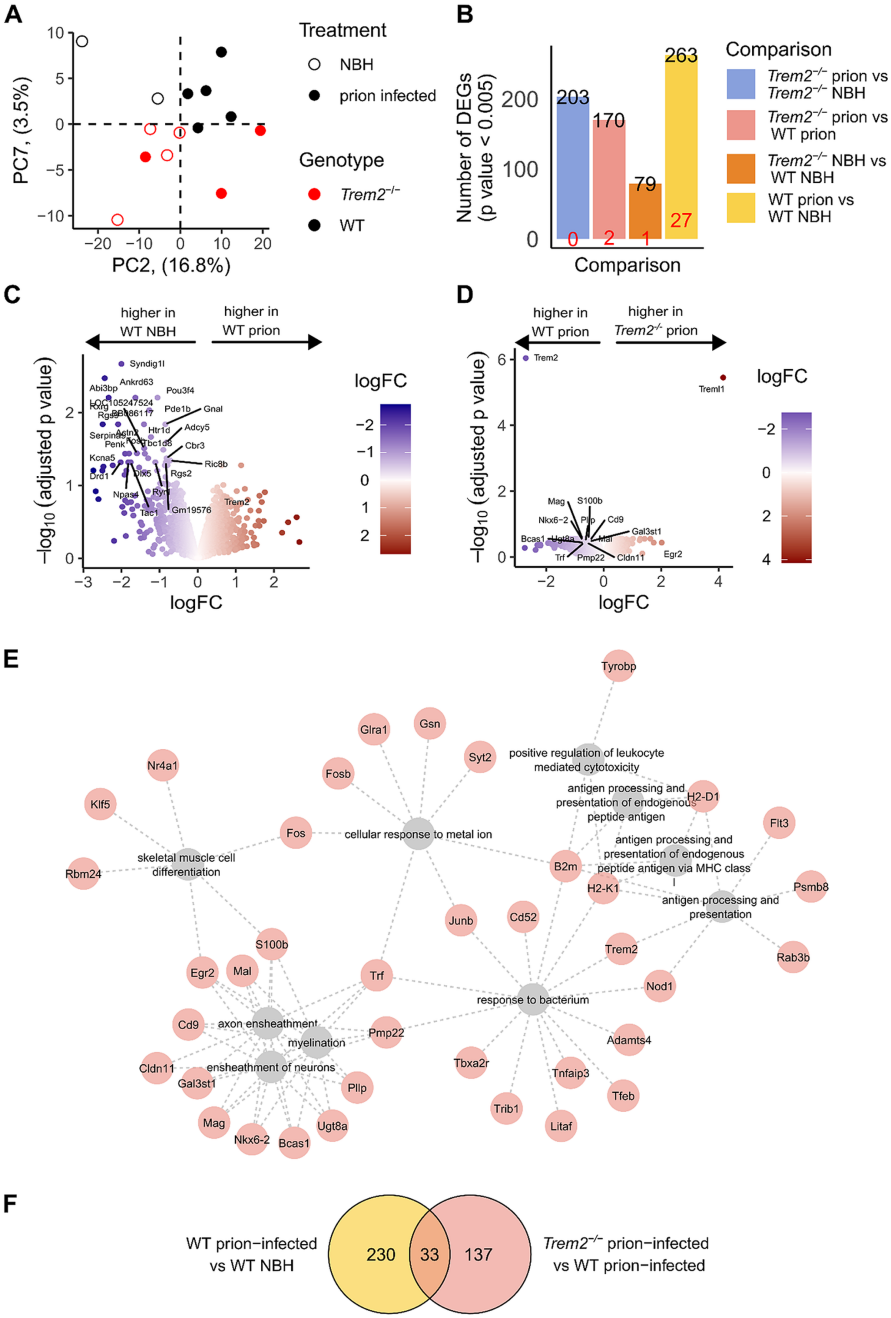
Transcriptional changes in the *Trem2*^−/−^ prion-infected disease model. **(A)** Principal component (PC) analysis allowed us to identify differences in treatment (NBH vs. prion-infected) along PC2, whilst we found segregation by genotype (*Trem2*^−/−^ vs. WT) along PC7. **(B)** Barplot indicates the number of differentially expressed genes with *p*-value <0.005 (indicated in black) and those with an adjusted *p* < 0.05 (red). **(C)** The volcano plot for the disease model signature shows the fold changes and corresponding adjusted *p*-values of WT prion-infected compared to WT-NBH mice. Genes with an adjusted p-value <0.05 have been labelled in addition to *Trem2*. **(D)** Volcano plot showing fold changes and corresponding adjusted p-values of *Trem2^−/−^* prion-infected vs. WT prion-infected mice. Genes with an adjusted p-value <0.05 have been labelled in addition to those genes annotated with specific GO terms (GO:0007272 ensheathment of neurons; GO:0008366 axon ensheathment; GO:0042552 myelination). **(E)** Gene network shows the top 10 enriched biological processes amongst the 170 DEGs (*p*-value <0.005) between Trem2−/− prion-infected and WT prion-infected mice, including processes of ensheathment of neurons, myelination, response to the bacterium, and antigen processing and presentation. **(F)** Overlap between disease signature and the effect of *Trem2*^−/−^ in the disease model.

Comparison of the global transcriptomes in each sample by principal component analysis showed that the samples segregated along the second principal component according to treatment (NBH vs. prion-infected samples; accounting for 16.8% of the variance), whereas the samples segregated by genotype (WT mice vs. *Trem2^−/−^* mice) along PC7 accounted for up to 3.5% of the explained variance (Figure 6A).

Differential expression analysis revealed 263 differentially expressed genes (DEGs) in the disease model (WT prion-infected vs. WT NBH; *p* < 0.005; Figures 6B,C, Supplementary Table S1). Gene Ontology (GO) enrichment analysis showed enrichment in genes associated with the G protein-coupled receptor signalling pathway (fold enrichment = 11.59, adjusted *p*-value = 3.478×10^−6^, *n* = 10) and other neuronal/synapse-related terms in genes expressed at lower levels in the prion-infected group, consistent with early indications of neurodegeneration (Supplementary Figure S1). To explore the effect of the *Trem2* genotype on prion disease, we compared the gene expression profiles of prion-infected *Trem2^−/−^* and prion-infected WT mice, identifying 170 DEGs (p < 0.005) (Figures 6B,D). Gene Ontology analysis revealed an enrichment of genes (e.g., *Mog, Mal, Pmp22, Mobp, and Mag*) involved in myelination (fold enrichment = 7.48, adjusted p-value = 4.80×10^−5^, *n* = 12) and ensheathment of neurons (fold enrichment = 7.97, adjusted p-value = 9.17×10^−6^, *n* = 13, Figure 6E, Supplementary Figure S1, and Supplementary Table S2). Interestingly, these genes were all expressed at lower levels in *Trem2^−/−^* prion-infected vs. WT prion-infected mice. Genes involved in myelination and axon ensheathment tend to have higher expression in the *Trem2*^−/−^ NBH group but decreased expression in *Trem2*^−/−^ mice inoculated with prion disease (*Mbp*, *Plp1*, *Mog*, *Mag*, *Mal*, and *Mobp*) (Figure 6D, Supplementary Figure S2, Supplementary Table S1). We overlaid the genotype DEGs from within the prion-infected condition with disease-altered DEGs in WT mice and identified 33 genes that were altered in both the disease (WT prion-infected vs. WT NBH mice) and genotype (*Trem2^−/−^* prion-infected vs. WT prion-infected mice) comparisons with an enrichment in antigen processing and presentation genes (Figure 6F, Supplementary Figure S2, Supplementary Table S3). In summary, whereas we observed a general global disease transcriptome response reflective of neurodegeneration in the brains of the prion-infected mice, in the absence of *Trem2,* the disease-induced gene expression involved in myelination and microglial/macrophage reactivity was blunted. We previously showed ME7 prion disease results in decreased Luxol fast blue myelin staining (Stables et al., 2024); however, we did not observe any differences in LFB staining between *Trem2*^−/−^ and WT prion-infected mice (Supplementary Figure S3), suggesting more subtle features of myelin structure/function may warrant investigation in the future.

## 4 Discussion

Inflammatory activity is now widely established as a feature of neurodegenerative diseases. The identification that genetic variants in *TREM2* are associated with an increased risk of AD and other neurodegenerative diseases, including PD and frontotemporal dementia (Rayaprolu et al., 2013; Cady et al., 2014), has highlighted the role of the immune response in influencing the pathogenesis of these diseases. To further understand the role of *Trem2* in the development of neurodegenerative disease, we used a well-established prion disease model, the ME7 scrapie strain, which, unlike many other disease models, develops overt neuronal pathology. Key findings are that the deletion of *Trem2* has no impact on PrP^d^ deposition after infection of the CNS with prions but that neuronal loss and vacuolation were increased in susceptible brain regions. This vulnerability was also associated with morphological and transcriptional changes indicative of blunted microglial reactivity in the brains of *Trem2*^−/−^ prion-infected mice. These findings are consistent with and substantially extend the previous studies by Zhu et al. (2015), who also showed that TREM2 deficiency did not alter PrP deposition in a different prion model. Our data, therefore, implicate TREM2-mediated microglial reactivity in protecting from neuronal pathology but independently of effects on misfolded prion protein deposition.

*Trem2* is predominantly expressed on microglia amongst CNS cell types and has a well-established role in the phagocytosis of apoptotic debris and the resolution of damage-associated inflammation (Takahashi et al., 2005). We show that the microglia morphological alterations from ramified to more amoeboid in prion disease are significantly attenuated in *Trem2*^−/−^ prion-infected compared to WT prion-infected counterparts, indicative of a less reactive phenotype at the clinical end-stage of disease despite no difference in clinical endpoint or disease progression. This is consistent with a previous study observing a lower density of microglia in mice infected with the Rocky Mountain Laboratories (RML) prion isolate, in which expression of TREM2 was upregulated 6.5-fold in the brains of infected mice (Zhu et al., 2015). Several other studies have reported a reduction in microglial density and/or other indices of their reactivity in animals lacking TREM2 (Zhu et al., 2015; Cantoni et al., 2015; Poliani et al., 2015) with less activated microglia observed upon cuprizone-induced demyelination (Cantoni et al., 2015) and in amyloidopathy and tauopathy models (Leyns et al., 2017; Vautheny et al., 2021; Meilandt et al., 2020), although the consequences of this may vary according to disease phase and context. Conversely, stimulating TREM2 signalling augments microglial proliferation and certain forms of reactivity in neurodegenerative disease models (Wang et al., 2020; Ellwanger et al., 2021; Cignarella et al., 2020). TREM2 is required for microglia to adopt certain disease-associated transcriptional states, notably characterised by high expression of genes, such as *Cst7, Spp1, Lgals,* and *Clec7a,* commonly referred to as disease-associated microglia/activated response microglia (DAM/ARM) (Keren-Shaul et al., 2017; Sala Frigerio et al., 2019; Hou et al., 2022), and observed in multiple disease and injury contexts. It is likely that the ability of TREM2 to regulate metabolic and bioenergetic adaptations at least partly underpins its role in reactive changes (Ulland et al., 2017). TREM2 has also been shown to be complexed with C1q in AD mice, and in the absence of TREM2, complement-mediated synapse loss is increased (Zhong et al., 2023).

The altered microglial reactivity we observed in *Trem2*^−/−^ prion-infected mice did not affect the distribution or magnitude of PrP^d^ deposition. Whilst TREM2 regulates phagolysosomal function, its effects on the deposition of misfolded protein in other models of proteinopathy are inconclusive and suggest that TREM2 and microglia likely influence neurodegenerative disease processes in proteinopathy through mechanisms beyond influencing misfolded protein deposition or clearance. Zhu et al. (2015) also saw no differences in prion accumulation in the brains of *Trem2*^−/−^ mice infected with RML prions, and our previous studies also showed that mice with a complete deficiency in microglia did not differ in the kinetics of prion accumulation in the brain when infected with ME7 scrapie prions (Bradford et al., 2022). In this regard, an important novel finding of our study is that neuronal loss was significantly greater in *Trem2*^−/−^ prion-infected mice in specific brain regions, thus suggesting that TREM2 promotes resilience against neuronal vulnerability in prion disease independently of affecting misfolded prion protein load. The concept of how microglia respond to abnormal protein deposition being critical to the downstream degenerative disease process, including neuronal degeneration, is increasingly recognised in the context of amyloid precursor protein (APP)-related amyloidopathy and supported by numerous risk genes for AD affecting the responsiveness of microglia and/or other innate immune cells (Hardy and Salih, 2021).

There are likely many ways in which TREM2, by influencing microglial reactive status, could modify neurodegenerative progression independently of prion protein load. Our transcriptional analysis performed at an early pre-clinical stage of the disease when PrP^d^ deposition has commenced but prior to overt vacuolation identified that genes changed by the *Trem2* genotype in the disease were enriched in those involved in the generation and maintenance of myelin. This is in agreement with previous studies reporting a link between TREM2 and myelination in overt de/remyelination models; abnormalities in remyelination upon cuprizone treatment have been reported in *Trem2^−/−^* mice, with *Trem2*-deficient microglia failing to remove damaged myelin (Poliani et al., 2015). *Trem2*^−/−^ animals have also been shown to be defective in the clearance of myelin debris, and more axonal pathology has been observed (Cantoni et al., 2015). A link has also been reported between axonal prion protein and myelin, with the loss of axonal prion protein triggering peripheral myelin loss (Bremer et al., 2010). A study using Myc-tagged PrP to characterise the interactome of the prion protein identified several proteins functioning in the maintenance of myelin (Rutishauser et al., 2009). Additionally, mutations in TREM2 cause Nasu–Hakola disease, characterised by demyelinating brain lesions (Paloneva et al., 2002). More broadly, microglia are implicated in the correct formation, maintenance, and regeneration of myelin (McNamara et al., 2023). Myelin provides trophic support to neurons and enables efficient action potential conduction. Further studies will be needed to establish the role that myelin-regulating properties of TREM2 and microglia have in prion and other neurodegenerative diseases that are not typically considered primary demyelinating disorders. Of note, we did not find differences in LFB staining, a measure of gross myelin status, between genotypes after ME7 prion inoculation, suggesting that more subtle properties of myelin health/function should be investigated in the future.

In summary, we conclude that loss of TREM2 results in altered microglial reactivity associated with increased sensitivity to prion-driven neuropathology, including vacuolation and neuronal loss that is independent of PrP^d^ deposition. TREM2 may control mechanisms downstream of exposure to prion protein that promote resilience to degenerative changes and further support TREM2 modulation as a rational therapeutic target in chronic neurodegenerative disease. Future studies to understand the signalling mechanisms connecting TREM2-mediated microglial reactivity with the altered health of other cellular components (e.g., taking advantage of single-cell resolution omics approaches) will be important to fully define how TREM2 can support neuroprotection in chronic disease.

## Data Availability

The datasets presented in this study can be found in online repositories. The names of the repository/repositories and accession number(s) can be found at: https://www.ncbi.nlm.nih.gov/geo/, GSE281022.

## Footnotes

^1^ https://imagej.net/ij/

^2^ http://www.affymetrix.com/support/technical/byproduct.affx?product=ht_mg-430_pm_ap

## Supplementary material

SUPPLEMENTARY FIGURE S1 Top enriched pathways among up and down regulated genes. Barplot indicates the top 10 enriched Gene Ontology biological processes among each set of differentially expressed genes. **(A)** Down regulated genes in Trem2-/- prion infected vs Trem2-/- NBH. **(B)** Down regulated genes in Trem2-/- prion infected vs WT prion infected. **(C)** Up regulated genes in Trem2-/- prion infected vs WT prion infected. **(D)** Down regulated genes in Trem2-/- NBH vs WT NBH. **(E)** Down regulated genes in WT prion infected vs WT NBH. **(F)** Up regulated genes in WT prion infected vs WT NBH. Dashed line indicates -log10 adjusted *p* value of 0.05.

SUPPLEMENTARY FIGURE S2 **(A)** Venn diagram showing overlapping DEGs in response to prion infection in WT and *Trem2^-/-^*. **(B)** Gene network shows the top 10 enriched biological processes among the 170 DEGs (*p* value < 0.005) between Trem2-/- prion infected and WT prion-infected, including processes of ensheathment of neurons, myelination, response to bacterium and antigen processing and presentation. **(C)** GO enrichment analysis among the set of 263 DEGs between WT prion-infected and WT NBH (*p* value < 0.005). Gene network shows the DEGs annotated in the top 10 enriched biological processes including G-protein coupled receptor signalling pathway.

SUPPLEMENTARY FIGURE S3 **(A)** Luxol fast blue staining in WT and *Trem2^-/-^* prion-infected mouse brain. **(B)** Quantification of luxol fast blue relative intensity; unpaired two-tailed t-test. Results are presented as mean ± SEM.
