# Supplementary figures and images for: TREM2 supports neuronal protection and microglial reactivity without an effect on misfolded protein deposition in chronic neurodegenerative prion disease
Source: Front Neurosci. 2025 May 7;19:1525017. doi: 10.3389/fnins.2025.1525017 (PMC12093410; doi:10.3389/fnins.2025.1525017)

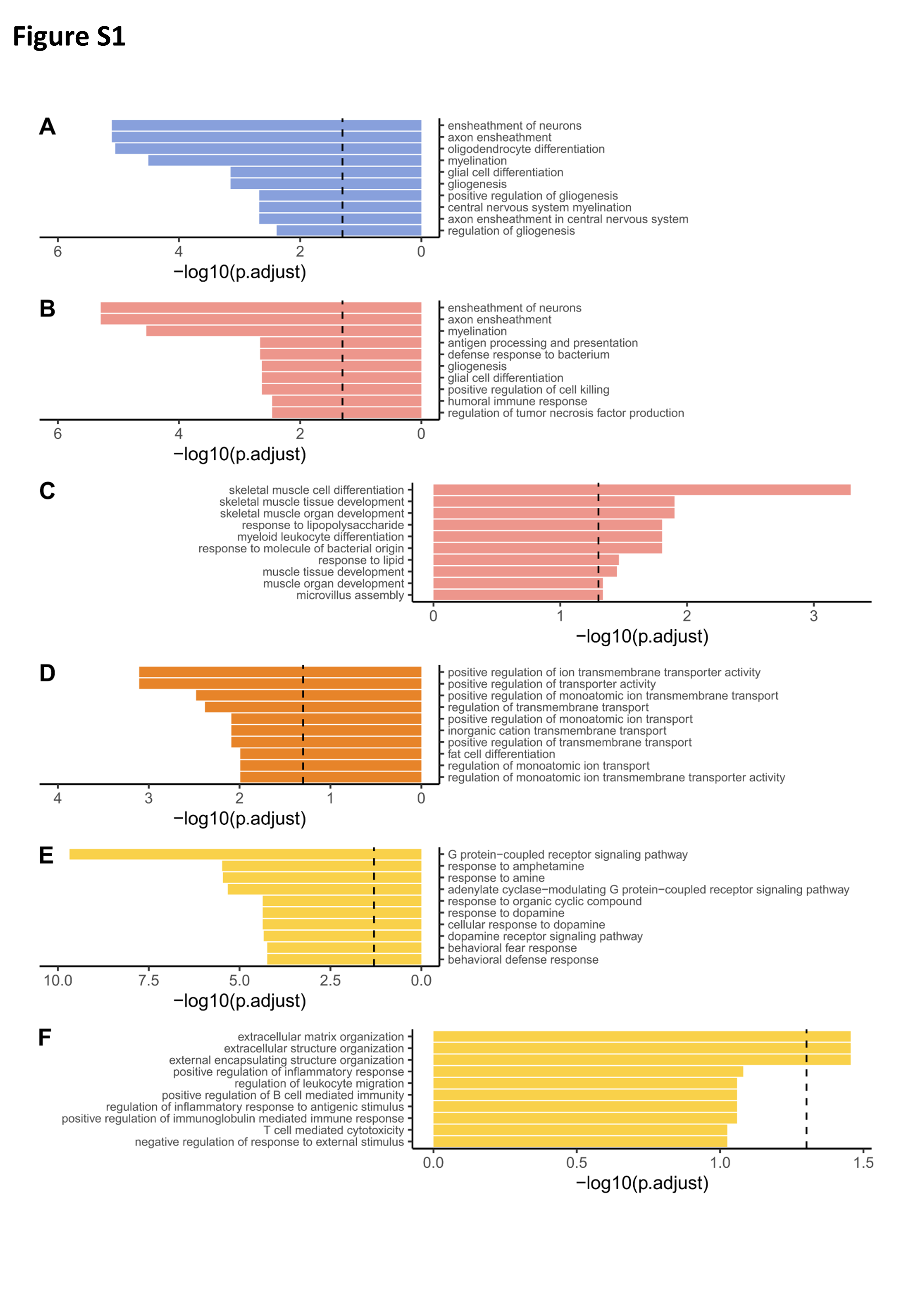

Supplement: SUPPLEMENTARY FIGURE S1 — Top enriched pathways among up and down regulated genes. Barplot indicates the top 10 enriched Gene Ontology biological processes among each set of differentially expressed genes. (A) Down regulated genes in Trem2-/- prion infected vs Trem2-/- NBH. (B) Down regulated genes in Trem2-/- prion infected vs WT prion infected. (C) Up regulated genes in Trem2-/- prion infected vs WT prion infected. (D) Down regulated genes in Trem2-/- NBH vs WT NBH. (E) Down regulated genes in WT prion infected vs WT NBH. (F) Up regulated genes in WT prion infected vs WT NBH. Dashed line indicates -log10 adjusted p value of 0.05. [file Image_1.TIF]

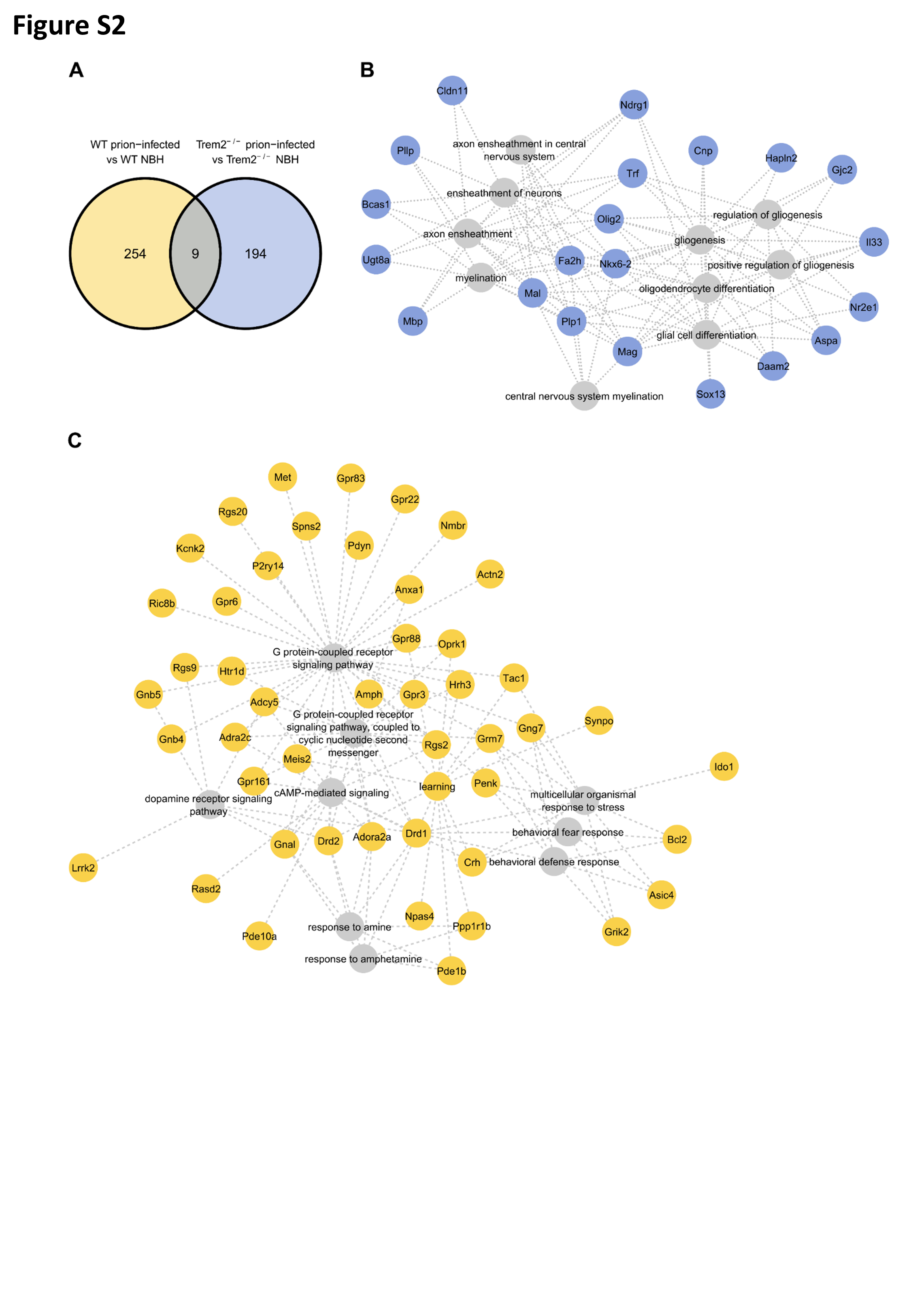

Supplement: SUPPLEMENTARY FIGURE S2 — (A) Venn diagram showing overlapping DEGs in response to prion infection in WT and Trem2-/-. (B) Gene network shows the top 10 enriched biological processes among the 170 DEGs (p value < 0.005) between Trem2-/- prion infected and WT prion-infected, including processes of ensheathment of neurons, myelination, response to bacterium and antigen processing and presentation. (C) GO enrichment analysis among the set of 263 DEGs between WT prion-infected and WT NBH (p value < 0.005). Gene network shows the DEGs annotated in the top 10 enriched biological processes including G-protein coupled receptor signalling pathway. [file Image_2.TIF]

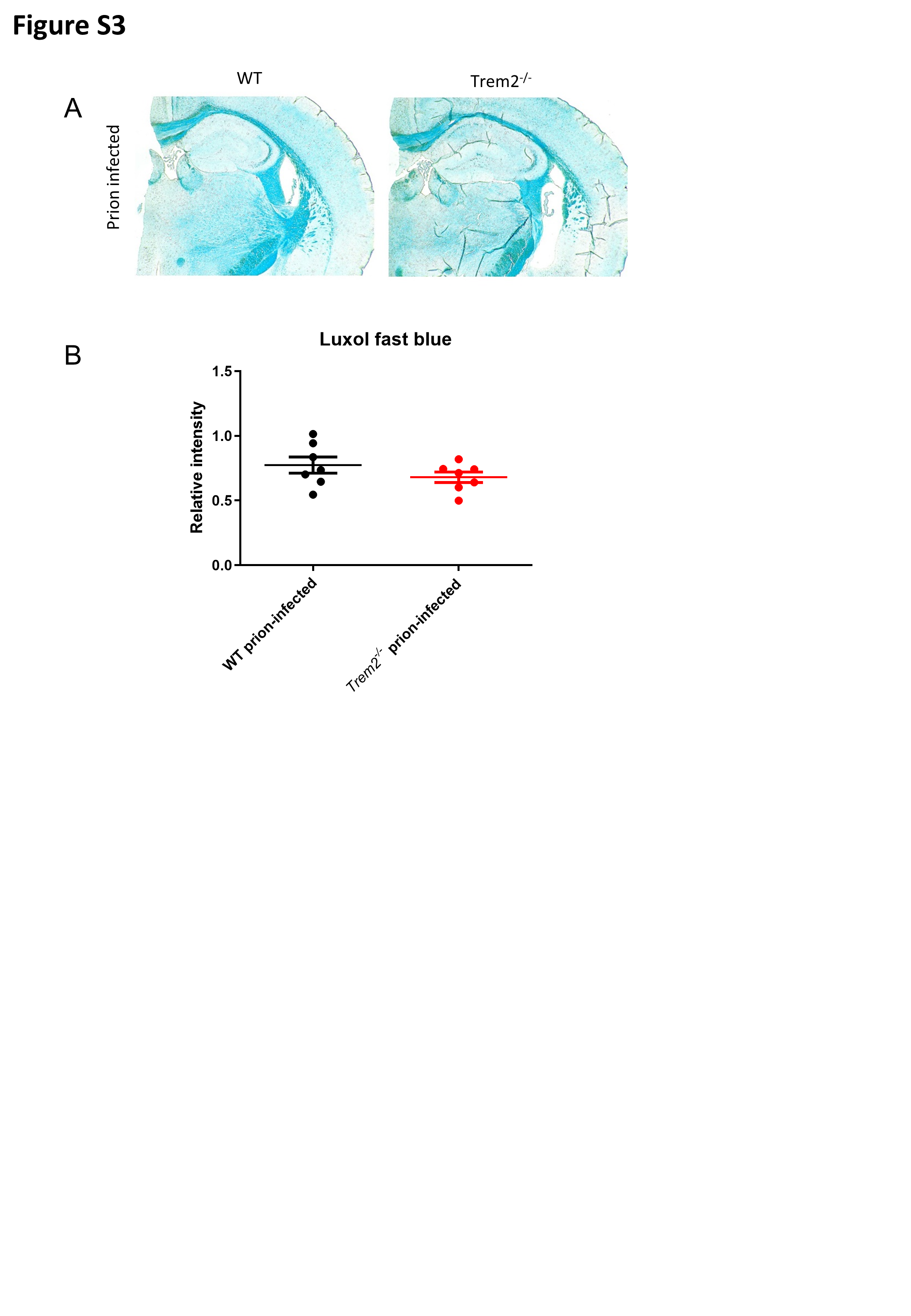

Supplement: SUPPLEMENTARY FIGURE S3 — (A) Luxol fast blue staining in WT and Trem2-/- prion-infected mouse brain. (B) Quantification of luxol fast blue relative intensity; unpaired two-tailed t-test. Results are presented as mean ± SEM. [file Image_3.TIF]
